# Combination Therapy Using Phytochemicals and PARP Inhibitors in Hybrid Nanocarriers: An Optimistic Approach for the Management of Colon Cancer

**DOI:** 10.3390/ijms26157350

**Published:** 2025-07-30

**Authors:** Mohammad Javed Qureshi, Gurpreet Kaur Narde, Alka Ahuja, Dhanalekshmi Unnikrishnan Meenakshi, Khalid Al Balushi

**Affiliations:** 1 Department of Pharmaceutics, College of Pharmacy, National University of Science and Technology, P.O. Box 620, Muscat PC130, Oman; 2 Department of Pharmacology and Biological Sciences, College of Pharmacy, National University of Science and Technology, P.O. Box 620, Muscat PC130, Oman

**Keywords:** PARP inhibitor, lipid hybrid nanoparticles, combination therapy, Olaparib, phytochemicals

## Abstract

DNA damage repair is a hallmark of any cancer growth, eventually leading to drug resistance and death. The poly ADP-ribose polymerase (PARP) enzyme is vital in repairing damaged DNA in normal and cancer cells with mutated *DNA damage response* (*DDR*) genes. Inhibitors of the PARP enzyme aid in chemotherapy, as shown by drug combinations such as Olaparib and Irinotecan in breast cancer treatment. However, the effect of Olaparib in colon cancer has not been studied extensively. Synthetic drugs have a significant limitation in cancer treatment due to drug resistance, leading to colon cancer relapse. Bioavailability of Olaparib and other PARP inhibitors is limited due to their hydrophobicity, which poses a significant challenge. These limitations and challenges can be addressed by encapsulating Olaparib in nanoparticles that could possibly increase the bioavailability of the drug at the site of action. New age nanoparticles, such as hybrid nanoparticles, provide superior quality in terms of design and circulatory time of the drug in the plasma. The side effects of Olaparib as a chemotherapeutic pave the way for exploring phytochemicals that may have similar effects. The combined impact of Olaparib and phytochemicals such as genistein, resveratrol and others in nano-encapsulated form can be explored in the treatment of colon cancer.

## 1. Introduction

**Figure 1:**
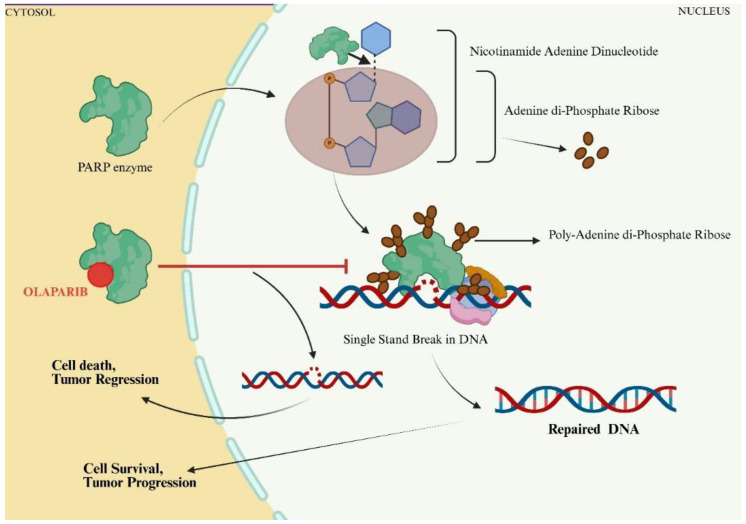
The PARP enzyme can repair DNA breaks using poly ADP ribose. However, in the presence of a PARP inhibitor like Olaparib (red), the enzyme is competitively inhibited and hence unable to repair the DNA break, resulting in cell death and eventually reducing the tumour size.

DNA damage happens constantly in humans due to external and internal factors such as reactive oxygen species (ROS) and ionising radiation. The DNA damage response (DDR) mechanism promptly produces the response to DNA damage. The mechanism involves transduction of several signals to form an appropriate response. The poly ADP-ribose polymerase (PARP) enzyme is essential to the DDR mechanism. The PARP1 enzyme is the most extensively characterized among the 17-member PARP enzyme family [1]. The PARP enzyme is involved in the repair of single-stranded DNA breaks (SSB) and double-stranded DNA breaks (DSB), as well as in base alteration and crosslinking. As shown in Figure 1, the PARP enzyme consumes NAD+ to generate ADP-ribose and nicotinamide. PARP enzyme has two distinct domains. The catalytic domain of PARP, like the ADP-ribosyltransferase (ART) enzyme, brings about the cleavage of NAD. The second domain is involved in DNA binding. It comprises Trp-Gly-Arg, which also acts as a regulator of the catalytic domain. PARP 1 also bears three zinc finger domains and a *BRCA-C* terminus domain, which helps in DNA binding [2]. The cleaved product, i.e., ADP-ribose, is added to DNA/RNA or proteins. The PARP 1 enzyme can add more ADP-ribose to elongate the first bound ADP-ribose into polymers. Once the poly ADP-ribose chain is attached to proteins, they are recognised by those with an ADP-ribose binding domain, which facilitates DNA repair [3,4]. Although DNA repair is an essential mechanism of cell survival, it becomes a liability in cancer cells and a hindrance in the treatment regimen.

The most mutated *DDR* genes in breast and ovarian cancer are *BRCA1* and *BRCA2*. There are other homologous recombination repair (HRR) genes, such as *ATM*, *ATR*, *CHK1*, *CHK2*, *DSS1*, *RPA1*, *NBS1*, *FANCD2*, *FANCA*, *CDK12*, *PALB2*, *BRIP1*, *RAD51B*, *RAD51C*, *RAD51D*, and *RAD54*, that are found to be mutated in several other types of cancers. These genes can no longer repair DNA damage in cancer cells from chemotherapy or radiotherapy [5]. However, the PARP enzyme takes over the role in repairing DNA damage in such cancer cells and studies have shown that the PARP enzyme plays a key role in base excision repair and two double-strand break (DSB) repair pathways, thereby supporting tumour progression [6]. Not only does the PARP enzyme repair DNA damage and help in tumour progression, but it also develops resistance to radiotherapy in cancer cells negative in *XRCC2* (X-ray repair complementing defective repair in Chinese hamster cells 2 gene) [7,8]. In such cells where DDR is damaged, PARP inhibitors (PARPi) can play a pivotal role as therapeutics. All PARP inhibitors are competitive inhibitors that mimic the nicotinamide moiety of NAD+ substrate, resulting in inactivation of PARP1 catalytic activity, pADPr synthesis, and DNA trapping with consequent stalling of the DNA replication fork and the formation of DNA breaks [9,10]. In such cancer cells, the strategy of inhibiting the PARP enzyme is used to target and destroy cancer cells. Drugs such as Olaparib, Niraparib, Rucaparib and Talazoparib are being used in the treatment of breast, ovarian, prostate and pancreatic cancers as PARP inhibitors [11].

The role of *DDR* and various genes in different cancers has been explored in recent years. In colorectal cancer (CRC), which is the third leading cause of death, the survival rate is only between 1 and 5 yr [12]. CRC is the third most common cancer worldwide, and according to estimates, by 2040 the burden of CRC will increase to 3.2 million new cases per year (an increase of 63%) and 1.6 million deaths per year (an increase of 73%) [13]. In CRC, the *DDR* gene, ataxia telangiectasia mutated (ATM), is one of the most commonly found mutated tumour suppressor genes. Normally, the ATM gene makes a protein in the nucleus, which helps control the rate of cell growth. It has been shown that mutated ATM expression is high in CRC tumours and serves as one of the biomarkers in CRC [14,15]. Olaparib, a PARPi, has been found to shrink the tumour size in the ATM deficient cell lines SK-CO-1 and HCT116 [16]. The treatment option of PARP inhibition is a promising strategy, but the associated side effects of any chemotherapeutic drug, such as drug resistance and relapse, are major deterrents. PARPi specifically disturbs the circadian metabolism and causes bone marrow suppression, as well as toxicity to all major human organs [17].

Interestingly, as alternatives, phytochemicals have been explored as potential anti-cancer agents [18]. Phytochemicals such as genistein and quercetin have been shown to activate p53 in ATM deficient cells, resulting in tumour regression [16,19]. Similarly, berberine, Physapubescin B, resveratrol, etc., have been shown to act as PARPi [18]. Synthetic PARPi such as Olaparib, Niraparib, etc., are administered orally, and after first-pass metabolism, the bioavailability is low. The PARPi are classified as Biopharmaceutical Classification System (BCS) class IV compounds (low solubility, low permeability). The bioavailability of Rucaparib is 30%, while for Olaparib it is 13% [20]. Phytochemicals that can act as PARPi are lipophilic, resulting in low bioavailability. However, encapsulation strategies using various lipids and polymers are used to overcome these limitations [21]. Much research has been done on lipid carriers for phytoconstituents targeting various diseases, including cancer. However, there is limited understanding of combining synthetic and phytochemical drugs for incremental efficacy as a therapeutic in CRC. The questions of how the combination helps in the pharmacokinetics and therapeutic potential of PARPi in minimizing the burden of anti-cancer drugs, and how it has an augmented impact in combating cancer, need to be explored further. This review article will discuss various synthetic chemotherapeutics and phytochemicals that act as PARP inhibitors, with a focus on encapsulation methods and their advantages and challenges. The article will also aim to underline the synergistic effect of synthetic chemotherapeutics and phytochemicals against CRC.

## 2. Synthetic PARPi Drugs and Their Combination in the Treatment of CRC

**Figure 2:**
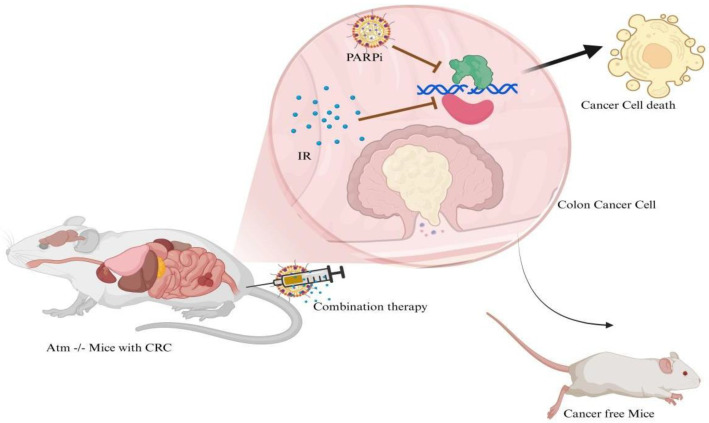
Mice with ATM gene mutation can be treated with a combination of Irinotecan and PARPi therapy.

According to Centers for Disease Control and Prevention (CDC), there are 25.4 million cases of colon cancer registered in US alone. This is an estimated number, but given the increasing demand of advanced treatment in cancer, the market potential for PARPi is huge, with a current valuation at USD 3.4 billion and a growth rate of 15.21% by 2031 [22]. Research has demonstrated that CRC is marred by defects in DDR machinery, such as ATM belonging to the phosphatidylinositol 3-kinase related kinases (PIKKs) family. It has been recorded in TOPARP-A phase II clinical trials that patients with *ATM* deficient cancers showed a positive response to the drug Olaparib [14]. Promising results have been obtained for PARPi drugs such as Olaparib in CRC cell lines. As reported by Changjiang, Olaparib was found to be cytotoxic in the *XRCC2* deficient colon cancer cell lines HCT116 and SW480. The results indicated that p53/p21 signalling pathways that promote senescence were increased by about five times in cells treated with Irinotecan (IR) and Olaparib in HCT116 and SW480 cells. The study showed that IR + Olaparib treatment led to significantly increased cellular senescence compared to either treatment alone, and the greatest senescence was in *XRCC2*-deficient cells. Similar results were obtained in vivo in *XRCC2* deficient tumours induced in mice. The tumours shrink following a 30-d drug treatment and showed increased radiosensitivity [23]. As demonstrated in Figure 2, ATM deficiency is established in CRC and research has indicated that PARPi are effective in treating such cancers. As shown by Chen, the *ATM* deficient CRC cell line SK-CO-1 was highly susceptible to Olaparib. At a 3 μM Olaparib concentration, 80–90% SK-CO-1 cells were found dead after 48 h. The important finding from this research was that HCT 116 cells that have low level of ATM protein showed resistance to Olaparib treatment. The promising concept is that about 18% of CRC patients have *ATM* deficiency, and hence the potential of PARPi in the treatment of CRC should be explored [24]. In an interesting clinical study performed on a 55-yr-old male patient, it was shown that, in *ATM* deficient stage IV CRC, a combination therapy of Olaparib-IR helped in reducing the tumour marker from 800 U/mL to 390 U/mL in the first course of treatment. The treatment was tolerated well and provided an option after all other avenues had failed [25].

Microsatellite instability (MSI) is present in 10–15% of CRC cases and is a marker for an underlying defect in mismatch repair (MMR). Research has shown that Niraparib, a PARPi, can elevate the impact of neoplastic drug SN-38 by an average of 2-fold in MSI cell lines [26]. In a similar study done by Pietro, it was identified that *ATM* mutations are a common genetic hallmark for treatment with PARPi. It was found that a combination of 50:1 ratio of Niraparib and IR showed a synergistic response in *ATM* deficient CRC cell lines. The in vitro results were corroborated by using the combination in mice xenograft models, using *ATM* deficient cells such as HCT116, SW480, and others. Interestingly, the ATM deficient mice xenograft models showed a significant tumour reduction of more than 90% and an extended survival rate of more than 2 weeks compared to the control groups [27]. Several mutations have been identified in CRC patients, of which *TRP53* (p53) has been identified in 92% cases and *ATM* and *BRCA*2 in 18% and 10% of cases respectively. In cases where the platinum-based drug treatment is exhausted, a combination therapy of PARPi and IR has been employed with some success. In one such study, it was shown that, in presence of Rucaparib PARP activity was reduced which may promote the effect of Irinotecan. In the CRC cell line RKO representing MSI mutation, a combination of IR:Rucaparib in a ratio of 1:2 showed 80% inhibition over a period of 48 h. In the presence of a combination of IR and Rucaparib, the expression of the PARP enzyme was reduced by four times in SW837 cells compared to IR treated cells. The in vivo analysis also showed that the inhibitory ratio of tumour weight when injected with Rucaparib + IR combination was 96%, while it was 80% and 79% in the Irinotecan and Rucaparib groups, respectively. The study concluded that a PARPi sensitises CRC cells for IR therapy in the presence of a PARPi [28].

In cancers where a single chemotherapeutic drug fails to provide desired outcome, combination therapies such as PARPi alongside other therapies like chemotherapy, radiation, or immunotherapy demonstrate significant potential. These combinations could improve the effectiveness of drugs, especially for difficult-to-treat tumours. Newer strategies, such as antibody–drug conjugates (ADCs), are being explored to leverage potential synergies while minimizing overlapping toxicities [29]. The encouraging combinations of PARPi currently being explored in human clinical trials, following successful preclinical results, include combining PARPi with drugs like Buparlisib, a PI3K inhibitor, Bevacizumab and Cediranib, which are anti-angiogenic drugs, Selumitinib, a MEK inhibitor, Durvalumab, an anti PD(L)1 agent, Ceralacertib, an ATR inhibitor, Adovacertib, a kinase inhibitor, and others [29].

In clinical trials, the results of treatment with PARPi such as Olaparib, Niraparib, Rucaparib and Talazoparib have been successful in breast and ovarian cancers [30,31,32]. For example, as shown by Erika in a clinical phase II study, a combination of Olaparib and Durvalumab proved to be effective in recurrent ovarian cancer. The study showed that, in a group of 35 patients, the expression of immunomodulatory molecules such as INFγ, with a 2.31 fold change, and chemokines such as CXCL9/CXCL10, with a 2.14 fold change, resulted from treatment with Olaparib and Durvalumab. The change in immunostimulatory environment was also correlated with progression-free survival of patients [33]. In a clinical trial performed on patients with advanced tumours with suspected homologous recombination deficiency (HRD) mutations, a combination therapy of 400 mg Rucaparib and 65–100 mg IR was given. All of the patients had previously been exposed to platinum therapy. Out of 15 patients, there 13 were available for evaluation of response of the drug. One patient showed a partial response, while five patients remained on the study for more than 6 mo. Three patients with ATM mutation survived for more than 1 yr in the study, while four patients showed some clinical benefits. The results of PARPi have been encouraging in advanced cancers. In CRC, the lab trials are convincing and holds promise for testing the efficacy of PARPi in CRC [34,35]. PARPi in combination with various anti-cancer drugs holds promise in the treatment of different cancers, including CRC. A more beneficial combination in which PARPi can be used is with phytochemicals, owing to their ability to help reduce the side effects of chemotherapeutics and alleviate drug resistance. In the next sections, we discuss the role of phytochemicals in CRC and their potential combinations with PARPi.

## 3. Promising Phytochemicals as PARPi in Treatment of Colon Cancer

**Figure 3:**
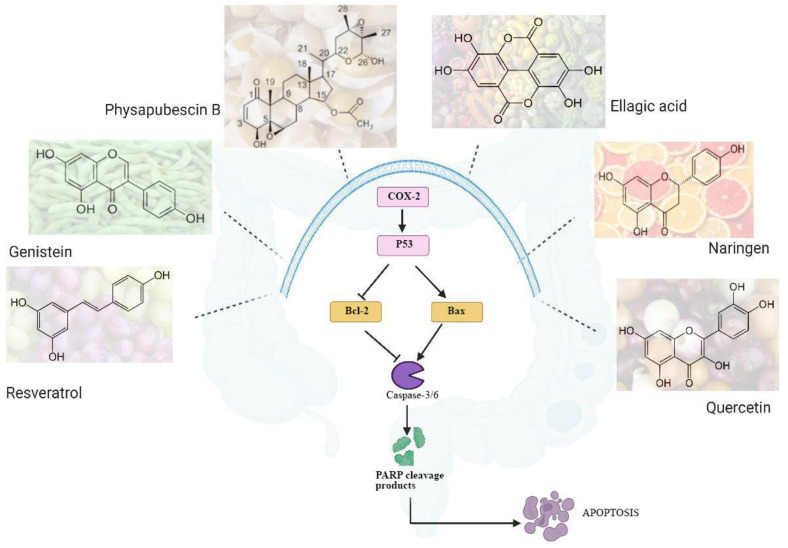
Different phytochemicals that can inhibit the intracellular caspase-3/6 activity in colon cancer, resulting in PARP enzyme cleavage, thus acting as PARP inhibitors. The result is inability of the treated cell to repair DNA damage, resulting in apoptosis.

Many phytochemicals, such as resveratrol, quercetin, and AKBA, are shown to have potent anti-cancer activity, particularly in CRC [21]. Such phytochemicals can be used effectively as PARPi if they can bind the active site of the enzyme which constitutes residues of Glu988 and the hotspot residues consisting of His862 and Tyr896 [36]. Molecular docking studies have shown −10 to −11 glide scores of ellagic acid, naringin, curcumin, and quercetin, which are comparable to those of Olaparib and Talazoparib. The pharmacophore modelling of Naringen and ellagic acid shows that there is a hydrogen bond interaction between the aromatic rings of phytochemicals and amino acids such as glycine, serine and glutamic acid present in the active site of the PARP enzyme model 5DS3. The study also highlighted that the complexes formed between the phytochemicals and PARP were stable, as the RMSD showed fluctuations of less than 0.4 Å, compared to 10 Å for the standard [37]. In a similar study performed by Mudassir, the docking scores for ellagic acid and genistein were found to be −10 and −10.5 respectively, whereas other phytochemicals such as quercetin, curcumin, resveratrol etc had scores below −10 [38]. Sridhar and his team analysed 0.2 million natural phytochemicals through computational studies and identified two potential PARP inhibitors. In the study, the compounds with docking scores ranging between −19.94 and −17.81kcal/mol were selected as the hit compounds and ADMET analysis was conducted for these hit compounds. The strong binding energies of −31.28, −25.64 and −23.14 kcal/mol with PARP enzyme helped to narrow the compounds down to three, HIT-1, HIT-3 and HIT-5. Finally, two compounds, HIT-3 and HIT-5, were selected as they could form complexes with strong affinities towards the PARP enzyme and, at the same time, satisfied all of the ADMET criteria. Furthermore, strong interactions were observed between compounds and Asp770, Ala880, Tyr889, Tyr896, Ala898, Asp899 and Tyr907 hotspot residues of PARP-1 [39]. These studies, in hindsight, provide a substantial clue that natural compounds could have a potential role in activating the PARP enzyme, either directly or indirectly. In the following section, the effects of each phytochemical that could be a potential PARP inhibitor are detailed. The empirical evidence of phytochemicals have provided evidence of increased amounts of PARP cleavage products via the Bax pathway, as depicted in Figure 3.

Genistein is a natural flavonoid found in soy beans and has potential applications as an anti-cancer drug. It has also been shown to increase the bioavailability of a drug by inhibiting the first-pass metabolism [40]. Genistein has been shown to induce cytotoxicity in cancer cells in vitro and to have a tumour regression effect in vivo. The mechanism by which genistein exerts its effect in vitro is by arresting the G2/M phase in the cell cycle and apoptosis through stress. At the molecular level, the stress was due to increase expression of caspases and other stress related proteins, resulting in apoptosis. Apoptotic cells were found to have increased levels of PARP cleavage products and other apoptosis related proteins, such as Bax. Genistein exhibited an indirect PARPi activity, and hence has a potential to be used in combination therapy with synthetic PARPi [18]. In CRC, genistein was found to exhibit dose-dependent cytotoxicity in SW480 and SW680 cells, with an IC50 of 280 μM and 667 μM, respectively. The production of reactive oxygen species was at least three times higher and, interestingly, the PARP cleavage was found to be almost double in genistein treated cells compared to untreated cells [41].

Ellagic acid is a phenolic lactone compound found in many vegetables and fruits. Owing to its strong anti-cancer properties, it was studied for its effect in the CRC cell line CCL-233. It was shown that ellagic acid had a concentration-dependent cytotoxicity effect on CCL-233 with an IC50 of 40 μM. The PARP activity was found to be 50% reduced in cells treated with 40 μM ellagic acid compared to control cells. This is compelling evidence that ellagic acid acts as a PARPi [42]. In a study performed by Ting, the impact of ellagic acid in sensitizing the CRC cell lines for 5-Fluorouracil (5-FU) was analysed. It was reported that inhibition of cell proliferation in HT-29 cells was almost doubled in the presence of a combination of 25 μM 5-FU and 25 μg/mL of ellagic acid when compared to the cell treatment performed with either alone. The enhanced inhibition was a result of expression of several apoptotic proteins, out of which cleaved PARP was also a major product, thereby linking the role of ellagic acid as a PARPi [43].

Naringen is a flavonoid found mostly in citrus fruits. The mechanism by which it exerts its anti-cancer effect is by inducing ER stress, leading to apoptosis. Naringen is a proven PARPi in vitro in cervical cancer C33A and breast cancer MDA-MB-468, as well as in colon cancer SW620 cell lines. The expression of Bcl-2–associated X protein (Bax) is upregulated, while the Bcl-2 protein is downregulated. This leads to increased expression of caspase-6 which cleaves the PARP enzyme [44,45,46]. In a study by Hun in different CRC cell lines, HCT116, SW480, HT-29, and LoVo were analysed for cytotoxicity induced by Naringen. In all the cell lines, concentration-dependent reduction in cell viability was observed. At 200 μM Naringen, the expression of cleaved PARP protein was found to be high. The study establishes that PARP cleavage is linked to high expression of ATF3 (activating transcription factor 3), which is responsible for apoptosis in colon cancer cells [47].

Resveratrol, a stilbene polyphenol abundantly found in grapes, is a well-established anti-cancer agent in CRC, as demonstrated by in vitro and in vivo analysis [21]. At the molecular level, it has been proved that resveratrol reduces the plasticity of colon cells by upregulating tumour suppressor protein p53 and downregulating PARP, thereby acting as a PARPi [48]. In several CRC cell lines, such as HCT116, SW480, SW680, HT29, COLO 201, it has been shown that resveratrol upregulates the production of PARP cleavage product through an increase in the Bax/Bcl-2 ratio [49]. Resveratrol occurs in two conformations, and in a thematic study, it was observed that the cis resveratrol actually acts as a PARPi. Interestingly, in physiological conditions, trans resveratrol is converted to cis resveratrol, which explains the anti-cancer effect of resveratrol [50]. It was shown that resveratrol exhibits dose-dependent cytotoxicity in the CRC cell lines HCT116 and Caco-2, with a IC50 of 170 and 120 μM, respectively. It was also proved empirically that the expression of cleaved caspase-9 and cleaved PARP protein increased in cells treated with 100 μM of resveratrol [51].

Quercetin is a flavonoid found in many vegetables and exerts its anti-cancer effect by several pathways. It can induce apoptosis by inhibiting caspase 3/6 and thus inhibits the PARP enzyme. As shown by Lin, quercetin induced apoptosis in HT29 cells in a concentration-dependent manner. At 200 μM, quercetin inhibited HT29 cells 5-fold more than in its absence. As a PARPi, it was shown that 200 μM quercetin increased cleaved caspase-3 [52]. Similar results were obtained in vitro in CRC cell lines such as HCT-15, Caco-2, SW480, CT-26 and DLD-1 as well as in vivo in rats [53,54,55]. In experimental Wistar rats with DMH induced colon cancer, quercetin pre-administration at 25 to 50 mg/kg body weight for 15 weeks resulted in reduced tumour growth, with restoration of redox balance. The levels of Cox-2 and iNOS doubled in rats that were pretreated with quercetin [56].

Physapubescin B is a steroidal substance isolated from *Physalis pubescens* L. (Solanaceae). In nude mouse models with prostate cancer xenografts, physapubescin B (50 mg/kg) decreased PC3 tumour growth by reducing the expression levels of *Ki-67*, *Cdc25C*, and full length PARP and increasing the apoptotic cell population within the tumour tissue [57].

## 4. Pharmacological Effects of the Combination of Synthetic and Phytochemical PARPi

PARPi are emerging as a very promising therapeutic option for cancer treatment; however, there are also associated side effects, such as fatigue, haematological and gastrointestinal effects. Grade 3–4 anaemia was observed in 19% of patients with Olaparib, 25% of patients with Niraparib, and 19% in patients with Rucaparib in a clinical study that involved 20 patients. Thrombocytopenia presented as haematological toxicity in more than a 60% patients and nausea of all grades was reported in almost all cases [58]. Phytochemicals have been shown to be potential antioxidants and anti-inflammatory molecules. It has been shown that crocin from saffron, gingerols and curcuminoids from the ginger family, and others can help alleviate the side effects of chemotherapy by reducing blood pressure, increasing high density lipoprotein (HDL) production, stimulating haematopoiesis and suppressing nausea and vomiting [59]. Not only do the phytochemicals reduce side effects associated with chemotherapy, but they also aids the treatment of cancer. A combination of phytochemicals and a synthetic PARP inhibitor drug can have a synergistic effect on cancer cells. As shown by Hou et al., berberine and Niraparib increased the apoptosis of tumour cells in ovarian cancer cell lines A2780 and HO8910. Berberine treatment of 10 to 20 μM for 48 h resulted in increased DNA damage, and with 10 μM of Niraparib, there was 90% inhibition in growth of HO8910 cells. In vivo experiments in mice with a A2780 xenograft also showed significant tumour size reduction when orally treated with 200 mg/kg berberine and 40 mg/kg Niraparib. Interestingly, it was found that the relative expression of the PARP enzyme was almost doubled in A2780 cells when treated with 20 μM berberine. Thus, a strategy of combining a PARPi like Niraparib obviously resulted in a synergistic effect of reduction in the PARP enzyme by at least 20% when compared to the control [60]. PARPi have gained relevance, as synthetic lethality molecules involving a pair of genes have an indispensable role in DNA repair, where one gene/protein has functional loss and the other is targeted in therapy. Although the concept of synthetic lethality began with *BRCA1*/*2* deficient cancer cells, it was extended to homologous repair (HR) deficient tumours. The detailed understanding of PARPi mechanisms have led to elucidation of several mechanisms of resistance to PARPi. For example, reversion of homologous recombination was the most common effect, apart from stabilization of the replication fork. The loss of the p53 binding protein (53BP1) shielding protein complex resulted in conferring resistance to PARPi [61,62]. Autophagy is also a mode of resistance that cancer cells exhibit for PARPi [63]. Upregulation of autophagy due to activation of the PI3K/Akt pathway results in inhibition of apoptosis in cancer cells. As shown by Alayev et al., the resistance to Rapamycin, an anti-cancer drug that inhibits the (mammalian target of Rapamycin) mTOR pathway, could be reversed in the presence of resveratrol. The breast cancer cells MCF-7, when treated with 20 nM Rapamycin and 100 μM resveratrol, reduced the Microtubule-associated protein 1A/1B-light chain 3 (LC3-II) expression levels by more than 2-fold. This indicated an inhibition in autophagy, as LC3-II serves as an autophagosomal marker. The same study also showed that PARP cleavage increased by 6-fold in presence of Rapamycin and resveratrol when compared to either of the pharmaceutics alone. Rapamycin in combination with resveratrol could kill MCF-7 cancer cells with double the efficiency [64]. In a similar study, it was shown that resveratrol could sensitize Talazoparib in targeting breast adenocarcinoma cells BMN673. In the study, it was shown that resveratrol causes disruption in the cell cycle, thereby enhancing the DNA damage by Talazoparib [65].

The aryl hydrocarbon receptor (AhR) ligand, a tumour suppressor gene when restored functionally, can act as an anti-cancer agent. Many phytochemicals have been reported to restore the AhR ligand activity in cancers such as breast cancer. In a study done by Jonathan, it was established that, apart from the PI3K/Akt pathway, a combination of AhR ligand and synthetic PARP inhibitor can be effective in treatment of triple negative breast cancer (TNBC) cells. A phytochemical derived from cruciferous vegetables, 3,3′-diindoylmethane (DIM), can enhance PARP activity in TNBC cells similar to 5F203 [66]. In a similar study, it was shown that DIM inhibited the growth of MCF-7 breast cancer cells with a IC50 of 20 μM. It was also found using immunoblot that DIM induced PARP cleavage and hence, with a PARPi, it can be very effectively used in treatment of cancer [66].

As reported by Saptarshi, resveratrol in combination with Olaparib resulted in downregulation of the HR pathway followed by accumulation of double stranded DNA breaks, ultimately leading to apoptosis in breast cancer cells. It was proven experimentally that the histone acetylase (HAT) activity reduced by 2-fold in MCF-7 cells treated with 25 μM resveratrol and 4 nM Olaparib compared to cells treated with Olaparib alone. HAT is responsible for acetylation of histone proteins that result in relaxation of the chromatin network. The study proved that resveratrol and Olaparib can have a synergistic effect in stopping DNA damage repair, resulting in apoptosis of MCF-7 cells [67]. All the studies summarized above indicate phytoconstituents’ protective and/or synergistic role in tandem with PARPi. Almost all the studies presented are targeting breast and ovarian cancer cell lines, since PARPi have been most extensively explored in cancers with *BRCA* 1/2 mutations. With the recent understanding of the molecular mechanism of PARPi, their role in other cancers, such as colorectal cancer, is being explored. The knowledge gaps in relation to the synergistic and/or protective effects of phyto PARPi in combination with synthetic PARPi targeting CRC need more research. Since the bioavailability of PARPi is low, advanced drug delivery methods using nanoencapsulation could be a way forward. The improved and advanced methods of PARPi delivery are discussed in the following section.

## 5. Nanoformulation Technology (LPHNP) Approach for PARPi in Cancer Treatment

The oral bioavailability of PARPi is a major challenge to effectively and safely delivering PARPi during clinical cancer therapy. While PARPi such as Talazoparib are currently being used in clinics for patients with breast and ovarian cancers harbouring the *BRCA* mutation (HR defective), the inherent and acquired drug resistance to PARP inhibitors by the restoration of *BRCA1*/*2* function (HR active) is the major challenge to successful therapy. The major reason for the development of resistance is the high dosage of drug given to overcome the bioavailability challenge. In order to address this issue, a drug delivery method using nanotechnology has been explored. Various nanoformulations allow drugs to be delivered via multiple routes, including oral administration, injection, and transdermal administration, which could change the current requirements for PARPi of a single administration. A multitude of PARPi delivery nanosystems are currently under investigation and are expected to exhibit excellent performance in future clinical therapy [68,69,70].

Guney Eskiler et al. produced Talazoparib loaded solid lipid nanoparticles (SLNs) and used treatments of 10 nM of Talazoparib with or without SLNs in the triple negative breast cancer cell lines HCC1937 (Talazoparib-sensitive) and HCC1937-R (Talazoparib-resistant) for 12 d [71]. The cell viability of HCC1937 reduced to 33.5 ± 1.4% and 29.0 ± 1.3 with Talazoparib and Talazoparib-SLNs, respectively. Surprisingly, the cell viability of HCC1937-R was nearly 100% and 34.3 ± 2.3% with Talazoparib and Talazoparib-SLNs, respectively. The results showed that there was no effect of Talazoparib in HCC1937-R cells after treatment, while Talazoparib-SLNs had the potential to overcome Talazoparib resistance in HCC1937-R cells. The ability of SLN loaded with Talazoparib to overcome drug resistance is because nanoparticles are known for controlled drug release, primarily at the site of action, thereby reducing the common pharmacological side effects of conventional drugs. Nanoparticles can also act as a protective sheath, minimizing off-target toxicities of the drug, modifying their cellular uptake, and reducing the likelihood of drug resistance development. Additionally, a dose of 10 nM of Talazoparib with or without SLNs in a normal human mammary breast epithelial cell line (MCF-10A) was added for 12 d to analyse the toxicity of the SLNs. The cell viability of MCF-10A cells treated with Talazoparib and Talazoparib-SLNs was 39.3 ± 1.5 and 63.5 ± 0.8%, respectively, indicating that SLNs are relatively safer carriers for cancer drug delivery. To improve the oral bioavailability, Pathade et al. formulated Olaparib loaded lipospheres by the melt dispersion method. This method was well suited to produce an optimum size of nanoparticles (128 nm), as particle size influences solubility, dissolution rate and oral bioavailability of the drug [72]. The oral bioavailability of Olaparib was investigated in Sprague Dawley rats following a single dose of oral administration of lipospheres. Interestingly, the half-life of Olaparib lipospheres was increased by 1.26-fold in comparison with Olaparib alone [73].

In another study, Baldwin et al. developed lipid-based nanoparticles loaded with Talazoparib that had a drug encapsulation efficiency of 76.9 ± 11.35% with the drug loading content of a therapeutic concentration of Talazoparib (153.8 ± 22.7 μg/mL). The therapeutic efficacy was tested in an intraperitoneal murine disseminated disease model. When three doses per week were administered by intraperitoneal injection, nano Talazoparib retarded disease progression and significantly reduced the formation of ascites. Unlike Talazoparib, nano Talazoparib showed low toxicity and prolonged half-life of drugs, contributing to a greater response rate with temozolomide in TC-71 Ewing sarcoma xenografts [74].

To augment the properties of drug delivery carriers in terms of high loading capacity and stealth properties, an advancement in nanotechnology is hybrid nanoparticles, where biodegradable polymers such as poly D,L-lactic-co-glycolic acid (PLGA), poly D,L-lactic acid (PLA), or poly 3-caprolactone (PCL) are used as a core of nanoparticle drug delivery vehicles. The polymeric nanoparticles have shown lower circulation half-life compared to their liposomal counterparts [75]. In order to increase the half-life of polymeric nanoparticles, a coating of lipid is used. The lipid layer also provides biomimetic properties and biocompatibility [76]. Lipid polymers plugged with PEG have been used to encapsulate therapeutic compounds. The PEG layer provides a stealth layer on the surface to avoid opsonization (Liposome-PEG 2013). These lipid polymer hybrid nanoparticles (LPHNP) have advantageous features, such as the capability of carrying highly hydrophobic drug with a high payload in their hydrophobic core, the possibility to attach functional groups to the hydrophilic shells that provide stearic protection, controlled drug release, and a longer drug half-life, thereby elevating the therapeutic efficacy [77].

In a study by Mensah et al., a formulation of Olaparib and cisplatin was prepared using the lipid bilayer approach to treat ovarian cancer. In order to increase the stability and pH sensitivity, the negatively charged liposomes were coated with polycation poly L-Lysine (PLL), which was layered by polyanion hyaluronic acid. The hybrid nanoparticles resulted in sustained release of the drugs in vivo. The Olaparib and/or cisplatin loaded nanoparticles did not result in any mortality of the mice models, which died within 8 d when treated with non-encapsulated drugs. The nanoformulation also reduced the side effects, such as <5% mild body weight loss and reduced myelosuppression, when compared to free drugs [78].

In another study, Talazoparib was encapsulated in lipid bilayers to test its efficacy against breast cancer. It was found that survival of mice induced with tumours increased from 52 d in free Talazoparib to 91 d in nano encapsulated Talazoparib. The nanoformulation of Talazoparib also resulted in 69% tumour regression, compared to only 21% in free Talazoparib. Weight reduction and alopecia were also minimal in the mice treated with nano Talazoparib compared to free Talazoparib [79].

With a similar approach, it was shown that an Olaparib nanoformulation can increase the impact of radiotherapy in p53^−/−^ prostate cancer cells. In mice models implanted with FKO1 cells, it was found that survival increased by 2-fold when a radiation dose of 10 Gy was given with nano Olaparib compared to when given alone [80].

**Figure 4:**
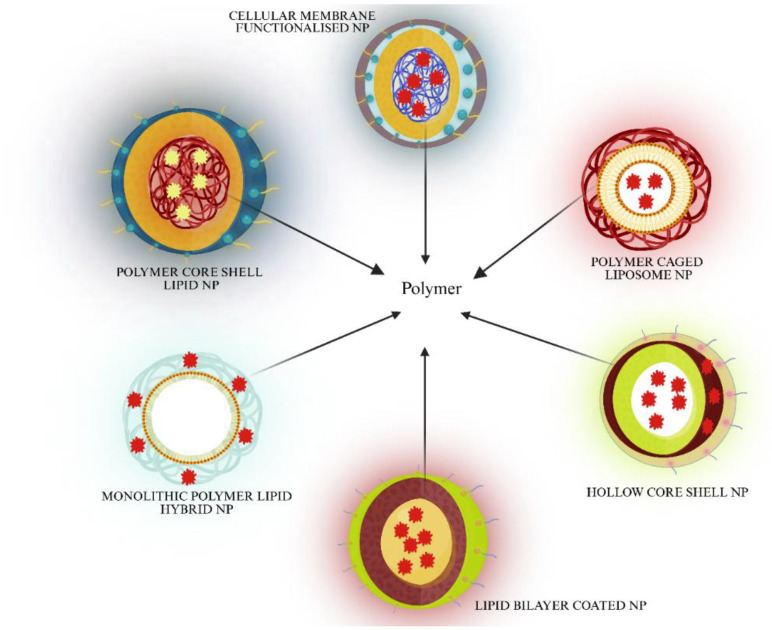
Different types of hybrid polymer–lipid nanoparticles that can carry the payload of hydrophobic drug molecules such as synthetic/phytochemical PARPi and effectively deliver the drug. In the figure, the drug molecule is shown in red, entrapped either in the polymer or in lipid. The lipid layers are shown in yellow/green as a core outer layer or in the form of a lipid bilayer. The outermost layer in most of the LPHNP is coated in lipid PEG, that is shown by a symbol with a tail. A hollow core is shown in white. In the cellular membrane functionalised nanoparticle, the grey colour represents the RBC layer.

As shown in Figure 4, various types of LPHNP that can be used in PARPi delivery, each with their own advantages and some limitations.

### 5.1. Polymer Core Lipid Shell Nanoparticles

This is the simplest form of polymer lipid nanoparticle, in which the core consists of polymer coated with a lipid mono or bilayer and the lipid PEG. The addition of lipids makes the nanoparticle biocompatible, as well as giving an advantage of polymer stability with sustained drug release. The core could also be made of an inorganic material, such as silica. The space between the polymer and lipid shell is occupied by an aqueous layer [81]. The lipid coating can be further coated with PEG lipids to enhance the stability of the core. Such polymer core nanoparticles with lipid shells have been effectively used in the treatment of lung cancer. One study showed a reduction in the tumour volume by four times when it was treated with polymer core lipid nanoparticles loaded with drugs such as paclitaxel and triptolide [82].

### 5.2. Lipid Bilayer Coated Nanoparticles

In this type of nanoparticle, the core is a lipid liposome. Since liposomes have difficulty in aggregating, coating them with a polymer ensures stability. Polymers such as dextran, polyglycerols, poloxamers, sialic acid derivatives, etc., can provide stability to the inner lipid core. Interestingly, the polymer can additionally be coated with a lipid of the opposite charge mostly associated with polyethylene glycol, thus imparting a longer circulation life [83]. However, a major disadvantage of this system is drug release, as the polymer coating over the liposomes becomes a barrier. In certain cases, the polymer can easily disintegrate, leaving the liposomes vulnerable to digestion by enzymes or the immune system [70].

### 5.3. Polymer Caged Liposome Nanoparticles

The limitation of liposomes to in aggregation is overcome by stabilizing them using a polymer in polymer cages liposomes. This strategy avoids drug leakage during the circulation time. The polymer cage can be made pH or protease enzyme sensitive, in order to release the drug effectively. However, in some cases, the polymer can degrade easily, thereby exposing the liposomes for degradation. Another limitation is the difficulty of such polymer caged nanoparticles in passing through the cell membrane [84,85]. A varied strategy is to adhere polymers or allow self-assembly of polymers on the liposomal surface [86].

### 5.4. Hollow Core Shell Nanoparticles

As the name suggests, these hybrid nanoparticles have a hollow core that is layered by a cationic lipid topped with a hydrophobic polymeric layer. The outermost layering is performed using a neutral molecule such as lipid PEG. The advantage of these hybrid nanoparticles is encapsulation of anionic molecules in the hollow core, and hydrophilic as well as hydrophobic molecules, given the structural layering of the nanoparticles [87,88].

### 5.5. Cellular Membrane Functionalized Nanoparticles

In order to mimic the natural membranes, fragments from cellular membranes are used to form vesicles, retaining the natural properties of the membrane. The most commonly used membrane is from RBC, and nanoerythrosomes have been studied extensively in passive targeting of drugs in tumours [83,89]. The main advantage is extended circulation times, but a major limitation is antigen compatibility on the membrane surface, which may cause immunogenic effects [90].

### 5.6. Monolithic Polymer–Lipid Hybrid Nanoparticles

These are also known as mixed lipid–polymer hybrid nanoparticles. The unique property of these nanoparticles is that the lipids are randomly dispersed, creating a core into which the hydrophobic drug can be loaded. The polymer forms the outer coating, thus making a colloidal carrier for drug distribution. These nanoparticles can be used to entrap extremely lipophilic drug molecules, such as retinoic acid, curcumin, etc. [91,92].

Since many studies, as mentioned above, indicate a robust response of PARPi in combination with other synthetic or phytochemical molecules, it is practical to design the formulation with the latest technology, in order to harness the maximum potential of combination drug therapy.

## 6. Challenges of Using PARPi in CRC

PARP inhibitors have shown promise in treating various cancers, including colon cancer, by targeting cancer cells’ DNA repair mechanisms. However, several challenges remain in using PARP inhibitors in CRC. The first challenge is the lack of biomarkers for patient selection. Identifying which colon cancer patients are most likely to benefit from PARP inhibitors is a significant challenge. While *BRCA* mutations and MSI status are predictive, they do not cover the full spectrum of patients [93]. Although ATM mutations in CRC have shown promising results with PARPi, there is no universally accepted biomarker to reliably predict response to PARP inhibitors in colon cancer, which can lead to suboptimal patient selection and treatment outcomes [94]. Secondly, as with all chemotherapeutics, PARPi also face the challenge of developing resistance and toxicity. Since PARPi target NAD+ cleavage, the non-specificity can result in unintended suppression of other NAD+ dependent enzymes and cellular functions, thereby elevating the risk of adverse effects [29]. Resistance can occur through secondary mutations that restore homologous recombination repair, increased drug efflux pump activity, or changes in the drug’s binding to its target. These adaptations can make PARP1 inhibitors less effective over time, highlighting the need for combination therapies to overcome resistance [95].

Another impact may comprise adverse reactions linked with PARP inhibition, such as myelodysplastic syndrome and acute myeloid leukaemia (MDS/AML), especially in patients harbouring a germline *BRCA* mutation. *BRCA1*, along with the Fanconi anaemia proteins (*BRCA2*), plays an essential role in DNA repair. Biallelic mutations of *BRCA2* are linked to Fanconi anaemia (AML), a genetic disorder characterised by congenital abnormalities and a significant increase in the risk of cancer predisposition [8,96].

Although there are no in vivo reports highlighting the impact of combining phytochemicals and synthetic PARPi, it would be worthwhile to study this. The challenge with phytochemicals is bioavailability, and hence formulating hybrid nanoparticles could address some of these challenges. Formulating PARPi with other phytochemicals/chemotherapeutics might provide promising results. The combination of PARPi with chemotherapy, phytochemicals, immunotherapy, or other targeted therapies encapsulated within nanoparticles may enhance therapeutic efficacy while mitigating resistance mechanisms. Furthermore, the formulation of hybrid nanoparticles could potentially overcome issues of bioavailability, and they may also help sustain the release of PARPi and improve tumour penetration. This approach could also pave the way for more personalised treatment, minimising adverse effects while enhancing overall response in CRC patients.

## 7. Conclusions

Combining PARP inhibitors with other treatments, such as chemotherapy, radiation, or immune therapies, shows great promise. These combinations could make the drugs even more effective, especially for tumours that are difficult to treat. Since phytochemicals hold significant promise in targeting colon cancer, a synergistic action with PARPi could augment the therapeutics of the treatment regime, while reducing side effects in parallel. The recent advancements in drug delivery, like hybrid lipid nanoparticles with reduced side effects and high payload of hydrophobic drugs, have emerged as a promising tool. Unlike breast and ovarian cancer, colon cancer cells are not deficient in *BRCA* genes. However other defective DNA damage response genes have shown an optimistic response towards PARPi therapy in CRC. Also, the ability of PARPi to modulate the immune response can pave the way to a more generalized approach of targeting different cancers. Cancer comprises a multitude of genetic variations compared to normal cells; hence, combination therapy must be an approach to combat cancer cells. Combining synthetic and phytochemical PARPi could be a fruitful way to treat complex cancers such as CRC, where other treatment regimens pose challenges.

## Abbreviations

The following abbreviations are used in this manuscript.

PARP	Poly ADP-ribose polymerase
DDR	DNA damage response
ROS	Reactive oxygen species
SSB/DSB	Single/double stranded breaks
NAD	Nicotinamide Adenine Dinucleotide
ART	ADP Ribosyl Transferase
BRCA 1/2	Breast cancer gene
HRR	Homologous Recombination Repair
CRC	Colorectal Cancer
ATM	Ataxia telangiectasia mutated
XRCC-2	X-ray repair cross-complementing 2
IR	Irinotecan
MMR	Mismatch repair
MSI	Micro satellite Instability
INFγ	Interferon gamma
CXCL9/10	C-X-C motif chemokine ligands
HRD	Homologous recombination deficiency
AKBA	Acetyl-11-keto-beta-boswellic acid
RMSD	Root Mean Square Deviation
5-FU	5-Fluorouracil
ATF 3	Activating Transcription factor 3
HDL	High density lipoprotein
AhR	aryl hydrocarbon receptor
DIM	3,3′-Diindoylmethane
HAT	Histone Acetylase
LPHNP	Lipid Polymer Hybrid Nanoparticle
SLNs	Solid lipid nanoparticles
PLGA	Poly D,L-Lactic-co-Glycolic Acid
PLA	Poly D,L-Lactic Acid
PCL	Poly 3-Caprolactone
PEG	Polyethylene glycol
